# Noncoding RNAs and Base Modifications: Epigenomic Players Implicated in Neurological Disorders and Tumorigenesis

**DOI:** 10.1155/2018/9016018

**Published:** 2018-09-24

**Authors:** Yujing Li, Changwon Park, Hong Jiang, Xuekun Li, Luciano Vellón

**Affiliations:** ^1^Emory University School of Medicine USA, USA; ^2^Xiangya Hospital at the Central South University, China; ^3^Zhejiang University School of Medicine, Hangzhou, Zhejiang 310029, China; ^4^Institute of Biology and Experimental Medicine (IBYME-CONICET), Buenos Aires, Argentina

Epigenetics is defined as the study of gene expression regulations by nongenetic but heritable other than DNA sequence variations. The epigenetic memories stored in the base modifications and histone modifications such as methylation, phosphorylation, and acetylation play important roles in regulation of all the metabolic pathways. Any aberrant alterations could lead to developmental abnormalities of diverse diseases including neurological disorders and cancers.

Before 2009, DNA methylation of 5-cytosine (5-mC) was the best characterized epigenetic marker. In recent years, functional significance of 5-hydroxycytosine (5-hmC), the sixth modified base, as an important epigenetic marker has been revealed. The 5-hmC, converted from 5mC and catalyzed by the ten-eleven translocation proteins (TETs), has become a hallmarker of cancer cells. The TET family consists of three members, TET1, TET2, and TET3, in mammals. It has been shown that TET1 functions as a tumor suppressor and tumor promoter in a cancer type-dependent manner. Deletion and inactivating mutations in the TET2 gene account for almost 30% of all myeloid malignancies, suggesting the essential roles of 5-hmC and TET2 in tumorigenesis. In addition, increasing accumulated lines of evidences are supporting the important roles of 5-hmC in almost all the known metabolic pathways that are involved in embryogenesis, cell reprogramming, stem cell self-renewal, proliferation and differentiation, central nervous system (CNS) development, and aging.

The regulatory mechanisms of 5-hmC in the metabolic pathways have been acknowledged to serve as the intermediate in the DNA demethylation process as well as the stable and independent marker to regulate gene expression by altering the epigenetic landscapes. Besides the base modifications, additional complexity of gene expression is added through dynamic changes on histone proteins and associated chromatin structural architectures. Although our understanding on the functions of epigenetic regulations is still incomplete, the accumulated data have suggested the link between aberrant alteration of base modification (such as 5-mC, 5-hmC, and m6A) levels and neurological diseases, cardiovascular diseases, and cancers. Additionally, noncoding RNAs (ncRNAs), particularly microRNAs (miRNAs) small RNAs and long noncoding RNAs (lncRNAs), have also been characterized as important epigenetic players in various pathophysiological events. Thus, it is of significance to publish a special issue focused on this topic. In this special issue, research and review articles are collected, covering the recent progress in the roles of the ncRNAS (particularly miRNAs and lncRNAs) and base modification epigenetic markers in pathogenesis of neurological disorders and cancers.

With the rapid development of high throughput sequencing technology and big data analysis, significant attentions have been paid to the identification of novel miRNA networks in regulating gene transcriptions involved in the pathogenesis of a certain type of disease such as cancer. Two research papers deal with the miRNA networks in breast cancer and renal cell carcinoma, respectively. Anda-Jauregui et al. systematically compared the highly influential nonredundant miRNAs named Commodore miRNAs (Cdre-miRs) from the healthy and cancerous breast tissues and found the significant variations in the degree, clustering coefficient, and redundancy distributions for the Cdre-miRs and their target genes involved in the metabolic networks for tumorigenesis. Furthermore, the analysis revealed five Cdre-miRs, including miR-let7i, miR-292b, miR-511, and miR-141, and each of which targets the genes involved in particular functions closely relating to tumorigenesis and metastasis.

In a separate report, Sage et al. identified a subset of novel miRNAs in kidney tissues by analyzing small RNA transcriptomes generated from the clear cell renal cell carcinoma (ccRCC) vs. the normal renal tissues. The expression differences of these novel miRNAs between the ccRCC and the nonmalignant renal tissues suggest that they could serve as specific markers of the kidney cancers. Furthermore, the authors found the significant association of the newly identified miRNAs with patient survival rates, providing a new resource for the renal cancer study and addressing the significance of exploration for the uncharacterized epitranscriptomes.

Long noncoding RNAs (lncRNAs) represent another large class of ncRNAs that regulate gene expression at a transcriptional level by targeting transcriptional activators or repressors, as well as at posttranscriptional levels by enhancing pre-mRNA processing, splicing, transport, translation, and degradation. Functionally, lncRNAs are involved in epigenetic modifications as well, leading to chromosome remodeling. Thus far, only limited numbers of lncRNAs were identified and characterized. A research paper by Long et al. reports the identification of a novel lncRNA from the model mice and detection of the significant difference in expression between WT and the SCA3/MJD model mice at the transcriptional level, opening a new avenue for dissection of the SCA3/MJD pathogenesis.

Despite extensive studies on the base modifications, the majority of the research has focused on the genomic DNA, limiting the availability of the epitranscriptomic information. Wei et al. compared 5-mC distribution in the epitranscriptomes of a human and mouse and found that the 5-mC was highly enriched at 5′UTRs of human and mouse mRNAs, and an inverse correlation between mRNA and DNA 5-mC levels at the CpG sites was observed. Generally, it is well known that 5-mC levels in genomic DNA are negatively correlated with transcriptional levels. In contrast, elevated RNA 5-mC levels enhance RNA translation and increase RNA half-life, although the detailed mechanisms remain elusive. Additionally, the 5-mC levels of the mitochondrial RNAs are significantly elevated compared to that of nuclear genomic DNA. Altogether, this study uncovers important roles of the 5-mC-based epitranscriptomic landscapes in metabolism of RNA particularly mRNA and mitochondrial RNA.

Significant efforts paid to the studies on the epigenetic involvement to the pathogenesis of tumorigenesis lead to the dramatic achievements in elucidating the mechanisms, contributing to development of novel strategies for the epigenetic-based therapeutic treatments of cancers. Two separate articles by Feng et al. and He et al. highlighted the recent progress in epigenetic regulation of cervical cancer and ovarian cancer, respectively. They focused on the roles of three base modification markers including 5-mC, 5-hmC, and 6-mA in DNA and/or RNA in regulation of genes/pathways involved in tumorigenesis.

To sum up, this special issue specifically addresses the research progress in base modifications and ncRNA-mediated epigenetic regulation of cancers and neurological disorders by either presenting original research outcomes or highlighting the recent advances in the related research fields. The authors of the research and review articles in the special issue hold the hope that they make a shortcut for the readers to get the related information easily, who are dedicated enthusiastically in the research field of epigenetic regulation of tumorigenesis and neurological diseases.

